# Factors Associated With Urolithiasis: A Hospital-Based Case-Control Study

**DOI:** 10.7759/cureus.37475

**Published:** 2023-04-12

**Authors:** Syed Owais, Mohamed Saif, Ahmad Omaid, Sofia Alfalasi, Anusha Sreejith, Muthana S Altaie

**Affiliations:** 1 Department of Community Medicine, College of Medicine, Gulf Medical University, Ajman, ARE; 2 Intensive Care Unit, Rashid Hospital, Dubai, ARE

**Keywords:** nephrolithiasis, risk factors, diet, urinary stones, urolithiasis

## Abstract

Background: Urolithiasis is a common preventable ailment. Previous studies showed that there are a lot of factors, such as dietary, health and environmental factors, which are likely to develop this condition. Only a few research have been conducted on urolithiasis in the UAE. Therefore, our study aimed to identify the factors associated with urolithiasis in the country, identify the symptoms of urolithiasis among the cases and identify the most common diagnostic methods.

Methodology: It was a case-control study design. The study population was adults above 18 years attending a tertiary care centre. Those who have confirmed diagnosis of urolithiasis and gave informed consent were considered cases and those who have not confirmed diagnosis of urolithiasis as controls. Patients with renal, bladder or urinary tract impairment or anomaly were excluded from the study. Ethical approval was obtained for the study.

Results: Crude odds ratio (OR) showed that age, gender, past treatment for urinary stones, and lifestyle factors such as diet and smoking are risk factors while exercising is a protective factor. Age-adjusted OR found that past treatment for the urinary disease (OR=10.4), consumption of oily food (OR=11.5), consumption of fast food (OR=11.0) and consumption of energy drinks (OR=5.9) were the significant risk factors for urolithiasis.

Conclusion: We found that past urinary disease treatment and diet are vital in developing urinary stones. Higher consumption of salty, oily, sugary and protein foods increases the odds of urinary diseases. Public awareness programs are essential to educating people about urolithiasis risk factors and preventive measures.

## Introduction

Urolithiasis is one of the critical public health problems and urologic diseases globally, with a prevalence ranging from 1% to 13%. According to a Global Burden of Disease (GBD) study, the cases and deaths due to urolithiasis have increased since 1999 [1,2].

Evidence shows that there are many risk factors for developing urolithiasis. Factors include age, gender, ethnicity, family history, past urinary diseases, dietary habits, physical activity, smoking and comorbid conditions like obesity [3-5]. High fluid intake is a critical protective factor recommended for decreasing the risk of urolithiasis. However, sodium overconsumption through soft drinks and animal proteins increases the urolithiasis risk [6].

Current scientific evidence clearly states the harmful effects of low fluid intake, high animal proteins and salt intake on the risk of stone formation. On the contrary, a diet with higher fluid intake is associated with the lowest risk for incident kidney stones. These observations, taken together, characterised the optimal diet for stone-forming patients and were confirmed by studies analysing the effect of different diets on kidney stone recurrence. Based on the available evidence, Mediterranean and vegetarian diets are the closest diets for optimal kidney stone prevention. In contrast, the Western diet, rich in meat and salt, brings the highest risk for stone formation [6]. It is recommended to increase the water intake and decrease sodium and animal consumption to reduce the lithogenic factors that would cause nephrolithiasis [7].

In another study, it was concluded that daily water consumption was substantially connected with urolithiasis, and the study determined that the minimum acceptable intake was 1.25 L/day. However, there was no statistically significant connection between the type of water ingested and stone formation [8]. Also, the chance of developing kidney stones was elevated among individuals with a genetic inclination who had infrequent urination throughout the day and consumed red meat at least once per month [9].

There is a noteworthy correlation between kidney stones and several factors, including being female, lacking education, having a body mass index (BMI) greater than 25 kg/m^2^, consuming more than 50 mg/L of sodium, drinking less than 1.5 L of water per day, obtaining water from a borewell, regularly consuming soft drinks, having a sedentary job and having a family history of kidney stones [10]. Similar results were found in other research that a positive correlation exists between a decreased dietary intake of fluids and an increased likelihood of experiencing a symptomatic kidney stone [11].

Urolithiasis is a public health issue in the Gulf with a high prevalence in the UAE. However, there needs to be more public awareness in the region despite the increasing prevalence with limited research done in the UAE. It was found that the populace had knowledge of nephrolithiasis but needed regarding the factors, like diet, that can cause the condition. Furthermore, if a person had a family history of urolithiasis, the risk of developing it was more than two times [12].

Several factors that contribute to urolithiasis differ between healthy individuals and patients. However, high BMI, insufficient fluid intake, excessive consumption of red meat and low levels of physical activity are the most influential factors in developing urolithiasis. To prevent urolithiasis, patients are advised to increase their daily fluid intake, engage in frequent and intense exercise and decrease the amount of red meat they consume [13].

Urolithiasis incidence is correlated with both workplace and environmental temperatures. Public health intervention techniques must be devised to stop people in high-risk neighbourhoods or vocations from being exposed to high ambient temperatures [14]. 

Urolithiasis is a common preventable ailment. Previous studies showed that there are a lot of factors, such as dietary, health and environmental factors, which are likely to develop this condition. Studies on urolithiasis in the UAE are limited [12]. Therefore, our study aimed to identify the factors associated with urolithiasis in the country, identify the symptoms of urolithiasis among the cases and identify the most common diagnostic methods.

## Materials and methods

The study followed a case-control design conducted in a tertiary care hospital in Ajman, UAE, between December 2020 and January 2021. The ethics approval was obtained from the Institutional Review Board of the University prior to the study. 

The sample size was calculated based on a study conducted in India, which reported that the percentage of controls exposed to food with a higher salt content is 25% and the odds ratio of 2.139 [15]. The calculation of the sample size was done using OpenEpi. The minimum sample size calculated was cases - 92 and controls - 184. The cases were selected from the Department of Urology for those who had confirmed the diagnosis of urolithiasis by ultrasound or radiographic procedure. The controls were those not diagnosed with urolithiasis and were selected from the general population. Patients with renal, bladder or urinary tract impairment or anomaly were excluded from the study.

A self-administered questionnaire was used to collect data from the participants after getting informed consent. Information related to demographic characteristics, information on previous urinary stones, family history of urolithiasis, and dietary and other lifestyle factors were included in the study. Anthropometric measures such as height, weight and BMI were also collected from the participants. Confidentiality, anonymity and privacy of the participants were maintained in the study. 

SPSS software version 27 (IBM Corp, Armonk, NY) was used for statistical analysis. A chi-square test assessed the association between the dependent and independent variables. Binomial and multinomial logistic regressions were used to determine the factors. A p-value less than 0.05 is statistically significant.

## Results

The total number of cases and controls collected in this study were 98 and 130, respectively. The association between socio-demographic characteristics and urolithiasis is given in Table 1. Age and gender were found to have statistically significant associations with urolithiasis. The proportion of cases among participants above the age of 30 was higher (75%) compared to the proportion of people between the ages of 18 and 20 (6.3%). A higher proportion (56.2%) of the males are cases compared to 27.4% of the females. BMI and nationality were found to be insignificant. However, the cases comprised more south-east nationals, such as India, when compared to the other nationalities.

**Table 1:** Association Between Socio-demographic Variables and Urolithiasis BMI: Body Mass Index; NS: Not Significant.

Variables		Controls N (%)	Cases N (%)	p-Value
Age group	18-20	74 (93.7)	5 (6.3)	<0.001
	21-30	40 (65.6)	21 (34.4)	
	>30	15 (11.6)	72 (73.5)	
	Total	129	98	
Gender	Male	53 (43.8)	68 (56.2)	<0.001
	Female	77 (72.6)	29 (27.4)	
	Total	130	97	
Nationality	Eastern Mediterranean	60 (56.1)	47 (43.9)	NS
	South-east Asian	52 (54.2)	44 (45.8)	
	Others	18 (72.0)	7 (28.0)	
	Total	130	98	
BMI	Normal	60 (63.8)	34 (36.2)	NS
	Overweight	59 (57.3)	44 (42.7)	
	Obese	7 (35.0)	14 (65.0)	
	Total	126	92	

Individuals who had treatment for urinary diseases (88.9%) had a significantly higher proportion of urolithiasis when compared to those who had not received any prior urinary disease treatment (34.9%) (Table 2).

**Table 2:** Association Between Family History and Details of Past Treatment of Urinary Diseases and Urolithiasis NS: Not Significant.

Variables		Controls N (%)	Cases N (%)	p-Value
Family history	No	90 (55.2)	73 (44.8)	NS
	Yes	40 (63.5)	23 (36.5)	
	Total	130	98	
Past urinary disease	No	109 (56.5)	84 (43.5)	NS
	Yes	21 (60.0)	14 (40.0)	
	Total	130	98	
Treated for urinary disease	No	123 (65.1)	66 (34.9)	<0.001
	Yes	4 (11.1)	32 (88.9)	
	Total	127	98	

The different diet consumption patterns of the participants are compared in Table 3. The individuals who consumed salty food daily (72%) and twice a week (32.6%) had significantly higher urolithiasis development proportion compared to those who did not or rarely consume (23%). Similar significant findings were present among participants who consumed fast food daily (45.1%) in contrast with those who consumed rarely or did not at all (28.7%).

**Table 3:** Association Between Diet and Urolithiasis NS: Not Significant.

Variables		Controls N (%)	Cases N (%)	p-Value
Consumption of salty food	Never/rarely	47 (77.0)	14 (23.0)	<0.001
	Twice a week	62 (67.4)	30 (32.6)	
	Daily	21 (28.0)	54 (72.0)	
	Total	130	98	
Consumption of red meat	Never/rarely	33 (66.0)	17 (34.0)	<0.05
	Twice a week	69 (50.0)	69 (50.0)	
	Daily	28 (70.0)	12 (30.0)	
	Total	130	98	
Consumption of fast food	Never/rarely	62 (71.3)	25 (28.7)	<0.001
	Twice a week	62 (54.9)	51 (45.1)	
	Daily	6 (21.4)	22 (78.6)	
	Total	130	98	
Consumption of energy drinks	Never	63 (61.8)	39 (38.2)	<0.001
	Rarely	56 (63.6)	32 (36.4)	
	Twice a week/daily	11 (28.9)	27 (71.1)	
	Total	130	98	
Amount of water they drink	1-3 cups	22 (44.9)	27 (55.1)	NS
	3-8 cups	87 (58.4)	62 (41.6)	
	More than 8 cups	21 (70.0)	9 (30.0)	
	Total	130	98	

A high proportion of individuals who ate red meat twice a day (50%) and daily (30%) had urolithiasis in comparison to those who did not or rarely ate it (34%). Twice a week/daily energy drink consumption (71.1%) was found to have a statistically significant increment in the development of urolithiasis compared to individuals who did not (38.2%).

Participants’ lifestyle behaviour and urolithiasis are associated in Table 4. The exercise was found to have a significant association with urolithiasis. Only 21.1% of participants who reported working out at least three times a week were cases compared to those who did not (62.3%). A significant association was also observed amongst individuals who smoked (76.6%) compared to non-smokers.

**Table 4:** Association Between Lifestyle Factors and Urolithiasis NS: Not Significant.

Variables		Controls N (%)	Cases N (%)	p-Value
Exercise	No exercise	46 (37.7)	76 (62.3)	<0.001
	A little	30 (62.5)	18 (37.5)	
	2 times a week	15 (78.9)	4 (21.1)	
	3 times a week	38 (100.0)	0 (0)	
	Total	129	98	
Smoking	No	72 (55.4)	58 (44.6)	<0.001
	Yes	11 (23.4)	36 (76.6)	
	Total	83	94	
Alcohol consumption	No	75 (45.7)	89 (54.3)	NS
	Yes	8 (61.5)	5 (38.5)	
	Total	83	94	

Binomial logistic analysis was performed to determine the crude odds ratio (Table 5). We found that the odds of urolithiasis in male participants were 3.4 times more than in females. Age also had a significant association with the prevalence of urolithiasis. Individuals older than 30 and 21-30 years were 71.7 times and 7.7 times, respectively, more likely to develop urolithiasis than those aged 18-20 years.

**Table 5:** Unadjusted Odds Ratio of Factors Associated With Urolithiasis CI: Confidence Interval; NS: Not Significant.

Variables		Unadjusted odds ratio	CI	p-Value
Age	18-20	1		
	21-30	7.7	2.7-22.1	<0.001
	>30	71.7	24.5-205.6	<0.001
Gender	Male	3.4	1.95-5.95	<0.001
	Female	1		
Past treatment for urinary disease	No	1		
	Yes	14.9	5-43.9	<0.001
Smoking	No	1		
	Yes	4.0	1.9-8.6	<0.001
Consumption of salty/oily food	Never/rarely	1		
	Twice a week	1.6	0.77-3.40	NS
	Daily	8.6	3.9-18.8	<0.001
Consumption of red meat	Never/rarely	1		
	Twice a week	1.9	0.99-3.8	NS
	Daily	0.83	0.34-2.0	NS
Consumption of fast food	Never/rarely	1		
	Twice a week	2.0	1.1-3.6	<0.05
	Daily	9.0	3.2-25.0	<0.001
Consumption of energy drinks	Never	1		
	Rarely	0.92	.51-1.6	NS
	Twice a week/daily	3.9	1.7-8.8	<0.001
Exercise	No exercise	1		
	A little	0.36	0.18-0.72	<0.01
	2 times a week	0.16	0.5-0.51	<0.01
	3 times a week	-		NS

We also noted a higher rate of risk, 14.9 times, amongst those previously treated for urinary diseases. Smoking also did seem to have a significant risk, with odds of four times for urolithiasis than those who did not smoke. 

The diet was significantly associated with the development of urolithiasis, with 8.6 times higher risk amongst those who consumed salt/oily food and nine times higher risk amongst daily fast-food eaters than those who did not or did, but rarely. Regular consumption of energy drinks twice a week/daily predisposed individuals to approximately 3.9 times higher risk for attaining urolithiasis than those who did not.

When considering an active lifestyle, individuals who exercised two times a week had 84% less chance of developing urolithiasis and 64% less chance amongst those who reported little regular exercise than those who did not exercise at all.

Multiple logistic regression gives the age-adjusted odds ratio for urolithiasis (Table 6). Past urinary disease treatment predisposes individuals to 10.4 times more risk of urolithiasis than individuals not treated previously for it. Consumption of salty/oily food daily increased the risk by 11.5 times and 11 times amongst individuals who consumed fast food daily compared to participants who did not or rarely consumed it, respectively. Twice a week/daily consumption of energy drinks incremented the risk of urolithiasis by 5.9 times as well. However, when adjusted for age, exercise did not significantly increase the risk of urolithiasis amongst the participants.

**Table 6:** Adjusted Odds Ratio of Factors Associated With Urolithiasis CI: Confidence Interval; NS: Not Significant.

Variables		Age-adjusted odds ratio	CI	Significance
Gender	Male	1.8	0.8-3.7	NS
	Female	1		
Past treatment for urinary disease	No	1		
	Yes	10.4	3.1-35.0	<0.001
Smoking	No	1		
	Yes	1.8	0.75-4.4	NS
Consumption of salty/oily food	Never/rarely	1		
	Twice a week	--	--	NS
	Daily	11.5	4.0-33.0	<0.001
Consumption of fast food	Never/rarely	1		
	Twice a week	2.8	1.2-6.5	<0.05
	Daily	11.0	3.2-37.3	<0.001
Consumption of energy drinks	Never	1		
	Rarely	--	--	NS
	Twice a week/daily	5.9	2.1-16.3	<0.001
Exercise	No exercise	1		
	A little	0.55	0.19-1.6	NS
	2 times a week	0.25	0.04-1.47	NS
	3 times a week	--	--	NS

## Discussion

Gender

Our study found that the male gender has a higher risk of developing urolithiasis than women. Similar results have been recorded by other studies as well. An observational cross-sectional study by Baatiah et al. to estimate and evaluate the prevalence of urolithiasis amongst the Saudi population found a 1.8 times risk for males compared to females. Males were more likely to develop urolithiasis [4]. Another retrospective study by Chien et al. showed that although men have a significantly higher risk of developing urolithiasis, females had a higher chronic kidney disease progression rate than their male counterparts [5].

Age

Our analysis found that individuals over 30 years had the highest prevalence of urolithiasis, followed by those between the ages of 21 and 30 years. A similar study by Chien et al. assessed 1802 patients and concluded that a higher age population (>60 years old) was 6.3 times more likely at risk of developing urolithiasis [5].

Another study by Shu et al. reported that men presented with more calcium oxalate (CaOx) and uric acid stones than women between the ages of 30-49 and 30-59 years. In the age ranges of 30-49 and 60-69 years, women contributed more infection and calcium phosphate (CaP) stones than males. Peak prevalence years were 50-59 for men and 60-69 for women. The lowest incidence was seen in adolescence for both genders. In both sexes, the prevalence of UA stones rose with age, whereas most infection stones declined [16].

History of urinary tract infection

Our study also found a significant correlation between the history of urinary tract disease and the development of urinary stones. Those with a urinary disease history are 10 times more likely to develop urolithiasis. Another study by Chien et al. showed similar results. They found that those previously treated for urinary diseases were 3.5 times more likely at risk [5].

A study by Andrew et al. concluded that bacteria and urinary stone diseases are clinically associated because they are often present in the same patient. And patients with urinary stone disease most often have a positive urine or stone culture [17].

Lifestyle 

When assessing the lifestyle of our participants, we found that individuals who smoked had a four times higher risk of developing urinary stones than those who did not. We also found a significant correlation between individuals who exercised regularly and those who did not. Individuals who exercised twice a week had 84% less chance of developing urinary stones and 64% less chance amongst those who reported little regular exercise than those who did not exercise.

A meta-analysis conducted by Ling et al. analysed five articles of 20,400 subjects. It found that individuals who smoked cigarettes had a significantly higher risk of developing urinary stones than those who never smoked [18].

Another research conducted in Saudi Arabia found an exponentially high risk of development of urolithiasis, by 70%, amongst individuals who smoked cigarettes compared to subjects who did not smoke, and a 45% increased risk among those who smoke hookah (a traditional water pipe) compared to those who do not smoke [4].

The UAE's arid climate predisposed individuals to dehydration and fluid loss. Research highlighting the country's temperature and working conditions found that such factors had a significant risk of developing urinary stones [14].

Diet

Diet is essential to consider when assessing the risk of developing urolithiasis. Our study showed that diet had a significant association in the development of urolithiasis, with ≈9 times higher risk amongst those who consumed salt/oily food and fast food daily than those who did not or did, but rarely. Regular consumption of energy drinks twice a week/daily predisposed individuals to a ≈4 times higher risk for urolithiasis than those who did not.

Similar results were reported by Rhu et al., who conducted a case-control study in Korea that showed that intake of non-diary animal protein predisposed individuals to 1.1 times more risk of urolithiasis [19]. Research conducted by Zhuo et al. showed that pickled food increased the risk of urolithiasis by 1.2 times in contrast with individuals who did not consume pickles that contained oil and salt [20].

## Conclusions

The study found that the risk factors of urolithiasis are the past treatment for urinary stones, and consumption of salty/oily food, fast food and energy drinks. We found that diet has a vital role in developing urinary stones.

The research findings highlight the importance of a healthy lifestyle, including a balanced diet and physical activity, in preventing urolithiasis. Public awareness programs are essential to educating people about urolithiasis risk factors and preventive measures. Future research should investigate the prevalence of urolithiasis and risk factors in the UAE population to develop effective preventive measures.
